# Field Performance of a Genetically Engineered Strain of Pink Bollworm

**DOI:** 10.1371/journal.pone.0024110

**Published:** 2011-09-13

**Authors:** Gregory S. Simmons, Andrew R. McKemey, Neil I. Morrison, Sinead O'Connell, Bruce E. Tabashnik, John Claus, Guoliang Fu, Guolei Tang, Mickey Sledge, Adam S. Walker, Caroline E. Phillips, Ernie D. Miller, Robert I. Rose, Robert T. Staten, Christl A. Donnelly, Luke Alphey

**Affiliations:** 1 Animal Plant Health and Inspection Service, Plant Protection and Quarantine, Centers for Plant Health Science and Technology, United States Department of Agriculture, Phoenix, Arizona, United States of America; 2 Animal Plant Health and Inspection Service, Plant Protection and Quarantine, Centers for Plant Health Science and Technology, United States Department of Agriculture, Moss Landing, California, United States of America; 3 Oxitec Limited, Oxford, United Kingdom; 4 Department of Entomology, University of Arizona, Tucson, Arizona, United States of America; 5 Frederick, Maryland, United States of America; 6 Medical Research Council Centre for Outbreak Analysis and Modelling, Department of Infectious Disease Epidemiology, Faculty of Medicine, Imperial College London, St Mary's Campus, London, United Kingdom; 7 Department of Zoology, University of Oxford, Oxford, United Kingdom; AgroParisTech, France

## Abstract

Pest insects harm crops, livestock and human health, either directly or by acting as vectors of disease. The Sterile Insect Technique (SIT) – mass-release of sterile insects to mate with, and thereby control, their wild counterparts – has been used successfully for decades to control several pest species, including pink bollworm, a lepidopteran pest of cotton. Although it has been suggested that genetic engineering of pest insects provides potential improvements, there is uncertainty regarding its impact on their field performance. Discrimination between released and wild moths caught in monitoring traps is essential for estimating wild population levels. To address concerns about the reliability of current marking methods, we developed a genetically engineered strain of pink bollworm with a heritable fluorescent marker, to improve discrimination of sterile from wild moths. Here, we report the results of field trials showing that this engineered strain performed well under field conditions. Our data show that attributes critical to SIT in the field – ability to find a mate and to initiate copulation, as well as dispersal and persistence in the release area – were comparable between the genetically engineered strain and a standard strain. To our knowledge, these represent the first open-field experiments with a genetically engineered insect. The results described here provide encouragement for the genetic control of insect pests.

## Introduction

Pink bollworm, *Pectinophora gossypiella* (Saunders), is the major lepidopteran pest of cotton in the southwestern USA. Since 1968 control measures have included SIT, which entails aerial release of radiation-sterilized pink bollworm moths to mate with wild pink bollworm and thereby reduce their reproductive potential [1], [2]. Currently, ∼200 million sterile moths are released each week during the cotton season as part of an area-wide eradication campaign that includes transgenic cotton expressing insecticidal proteins from *Bacillus thuringiensis* (*Bt* cotton), mating disruption with synthetic pheromones and other control measures [3], [4]. Moth populations are monitored using sticky traps with a pheromone lure [3]. In order to monitor levels of wild moths, and to assess the recapture rates of sterile moths, a method is required to distinguish between these two types of moth, which are normally indistinguishable even by microscopic examination.

Current practice is to add a lipid-soluble red dye, ‘Calco Red’ (Oil Red 2144, Royce International), to the larval diet, which imparts a red color to the fatty tissues of the adult moth [5]. This red coloration is clearly visible in most trapped moths but a weakly stained captured moth can also be homogenized and evaluated with a more sensitive chromatography test. However, anecdotal field experience suggests that a small fraction of sterile moths do not retain sufficient dye in their tissues to give this positive signal [6], [7]. This is a significant problem when the detection of a single wild moth can lead to a control or regulatory response [8]. If that ‘wild’ moth were in fact a mis-identified sterile moth, this effort and expense would be wasted. Another potential source of undyed sterile moths is the F_1_ progeny of released sterile moths that mate with wild moths (or even other sterile moths). The radiation dose used for pink bollworm SIT does not provide 100% sterility, being a compromise between a higher dose to give more complete sterility and a lower dose to minimize the radiation damage and consequent loss of performance of the sterile insects. For moths, F_1_ progeny of insects irradiated at sub-sterilizing doses are themselves sterile [9] – an effect known as F_1_ sterility – and therefore properly classified as sterile rather than wild moths, even though they developed from eggs laid in the field. These F_1_ sterile moths will not be stained with Calco Red and therefore form a second potential class of undyed sterile moths.

An easily scored heritable genetic marker would be useful, either to replace the Calco Red or as an independent backup. Transgenic moths expressing a fluorescent protein have this potential, when the modification can be accomplished without associated performance losses that outweigh the benefit. Whether that is achievable is unclear, as transgenesis is expected to impose some level of fitness cost [10]–[13]. Here we describe the development of such a fluorescent-marked strain, OX1138B, and direct comparison of this strain with the standard SIT strain (‘APHIS’) in large-scale open field experiments.

## Results

**Figure 1:**
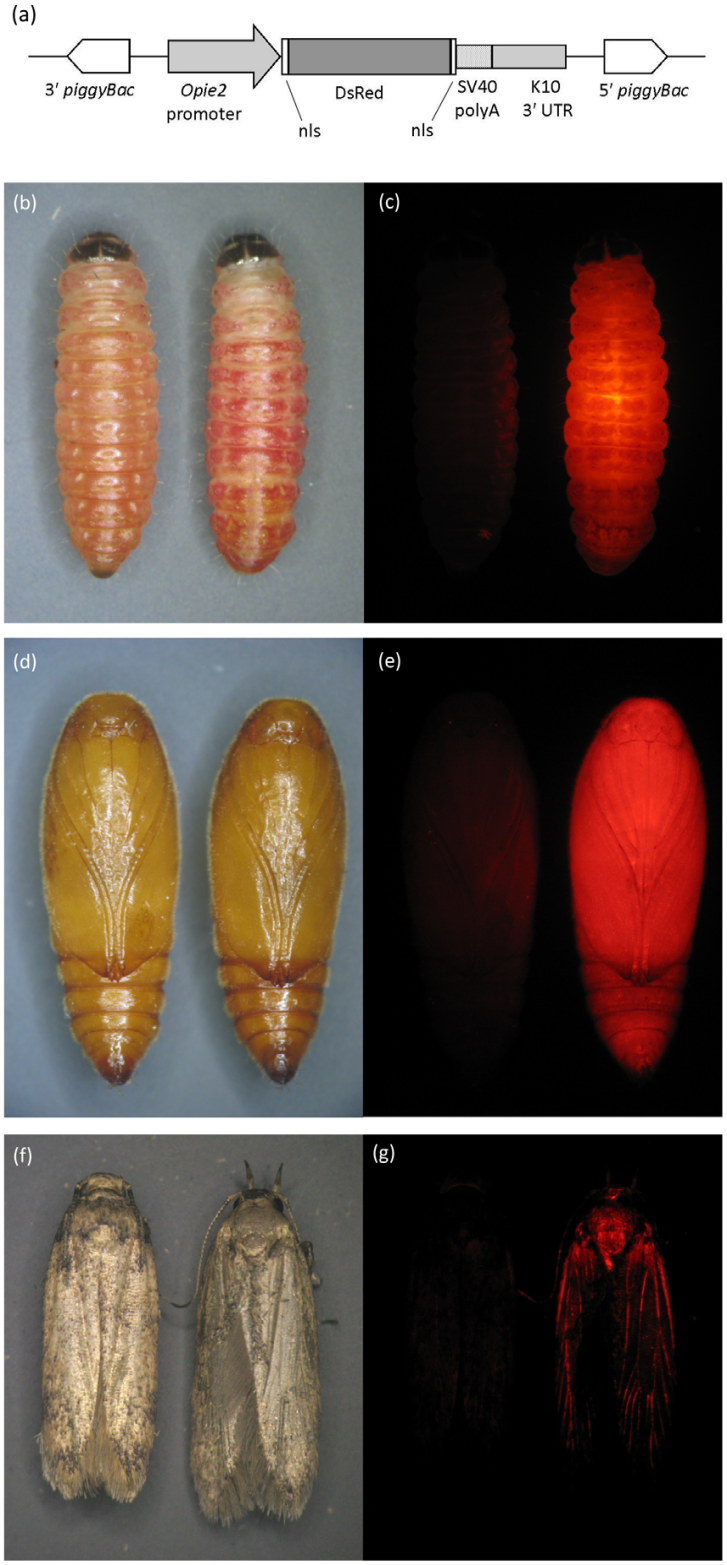
The OX1138 construct and the phenotype of the OX1138B strain. (a) Diagram of the OX1138 construct, showing its functional components (nls, nuclear localization signal; see also Materials and Methods); DsRed2 fluorescence in final instar wild type (left) and OX1138B (right) larvae, shown under bright field (b) and DsRed2 excitation wavelength light (c); DsRed2 fluorescence in wild type (left) and OX1138B (right) pupae, shown under bright field (d) and DsRed2 excitation wavelength light (e); DsRed2 fluorescence in wild type (left) and OX1138B (right) adults, shown under bright field (f) and DsRed2 excitation wavelength light (g).

By microinjection of DNA into the APHIS strain, we constructed a transgenic derivative strain, OX1138B, which expresses DsRed2, a red fluorescent protein (Clontech Laboratories, Inc.) [14], [15]. This allows OX1138B moths to be identified by fluorescence microscopy (Figure 1).

**Figure 2:**
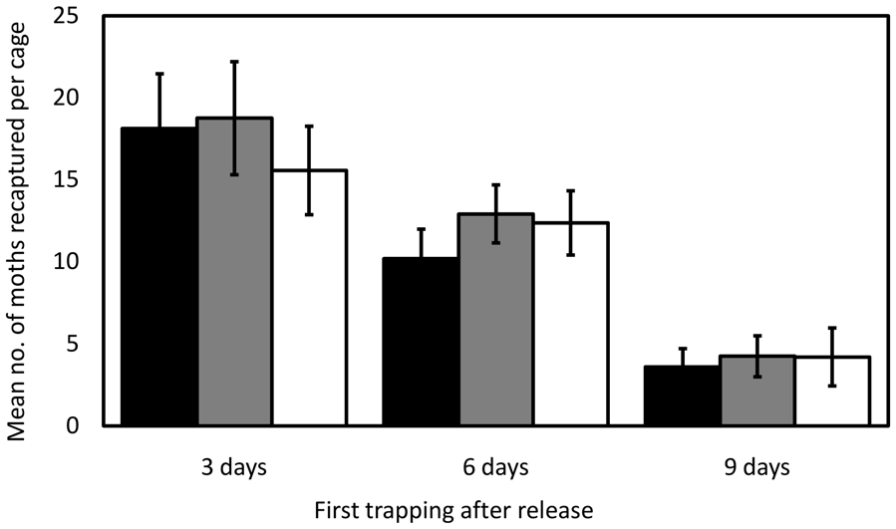
Mean recapture of three moth types in cages with pheromone-baited traps first set 3 days, 6 days or 9 days after release. Black bars = OX1138B 100 Gy; grey bars = APHIS 100 Gy; white bars = APHIS 200 Gy; error bars indicate Standard Error of Mean. There were no significant differences in recaptures between the groups on the first day of trapping or total recaptures (logistic regression model, p = 0.07 and p = 0.10, respectively).

In 2006 experiments, three moth treatment types–OX1138B gamma-irradiated at 100 Gy, APHIS irradiated at 100 Gy and APHIS irradiated at 200 Gy–were co-released in cages. Their performance–pheromone response and persistence–was tested to look for differences between OX1138B and APHIS, and to assess whether a reduced radiation dose (from 200 Gy to 100 Gy) provided clear performance benefits. Each moth type was marked as pupae with a different fluorescent powder (DayGlo Color Corp.) and the resulting adults irradiated at the appropriate dose. Fifty male moths of the three moth types were co-released in a field cage with cotton plants. Traps baited with gossyplure, a synthetic form of the female pink bollworm sex pheromone, were placed either 3, 6, or 9 days following release to look for differences in longevity between the moth types. The pupal weights of collections of both strains were measured to assess the relative quality of APHIS and OX1138B moths (APHIS 100 Gy and 200 Gy were taken from the same pupae collections): OX1138B pupae were significantly smaller than APHIS pupae collected for adult releases where traps were first set after 3 and 9 days (two-way Kruskal-Wallis test; p<0.01 and p = 0.02, respectively), with a similar non-significant trend where traps were set 6 days after release (p = 0.09). These differences were likely due to small variations in rearing conditions between the strains at different times, and reduced pupae size would be expected to have a negative effect on performance, if any effect. The moth type of recaptured males was identified by screening for fluorescent powder color, and for the presence or absence of the DsRed2 marker. This was undertaken in traps collected daily over the subsequent 1–2 weeks. Analysis by a logistic regression model showed that recaptures were not significantly different between the groups, either in terms of numbers caught on the first day of trapping (logistic regression model, p = 0.07) or total recaptures (p = 0.10) (Figure 2).

These field cage experiments showed no obvious performance defect in OX1138B relative to the APHIS strain, despite their smaller size in some cages. Preliminary field releases were also conducted in 2006 to develop and refine procedures for subsequent open field trials. We therefore decided to compare the performance of the two strains under field conditions. For field comparison with the APHIS strain, moths were reared in a quarantine facility using diet, equipment and methods equivalent to those of the main SIT program. APHIS strain pupae were obtained from the pink bollworm mass-rearing facility (MRF) and allowed to emerge as adults in the quarantine facility, in parallel with the OX1138B moths. Standard measures of strain and rearing quality were compared between the two strains. For the pink bollworm SIT program, moth weight is used as a measure of quality. The flight ability of pink bollworm, for example, correlates with pupal weight [16]. A slight difference in, for example, the density of larvae in diet can lead to differences in pupal size. Mean weights of adult moths were calculated from samples taken regularly from the two release streams. Over the course of the strain-comparison experiment, there was not a significant difference in the mean weight of OX1138B and APHIS adults: 7.78 mg (95% CI: 7.68–8.04), compared with 7.90 mg (95% CI: 7.59–8.15), respectively (p = 0.34). In addition, the rate of post-eclosion mortality in the two groups −0.80% (95% CI: 0.25–1.36%) in OX1138B moths, 1.2% (95% CI: 0.61–1.69%) in APHIS moths-was considered appropriately low with no significant difference between the strains (p = 0.08). These findings indicate that rearing and handling conditions were closely equivalent and also that there were no gross intrinsic differences between the strains based on these parameters. Moths were sterilized by irradiation (200 Gy) and the two strains mixed in equal proportion before transfer to the field site.

**Figure 3:**
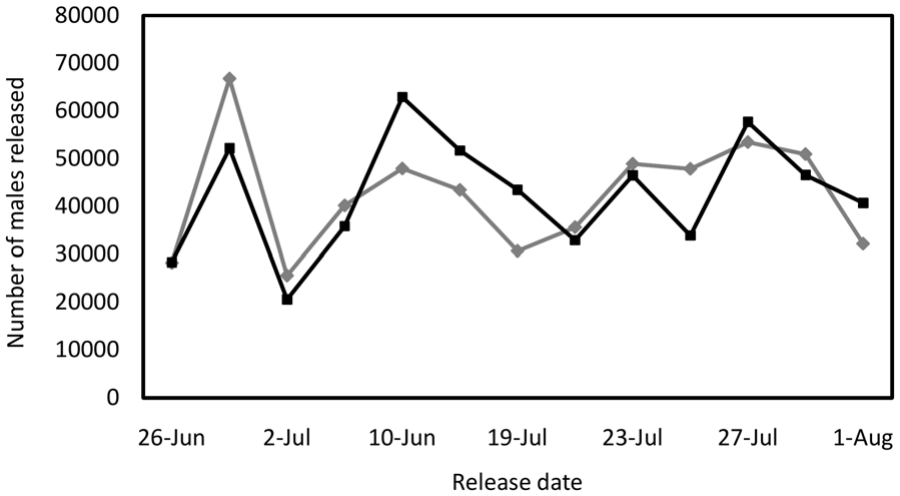
Number of OX1138B and APHIS male moths released over all fields during the trial period. Black line = OX1138B; grey line = APHIS.

**Figure 4:**
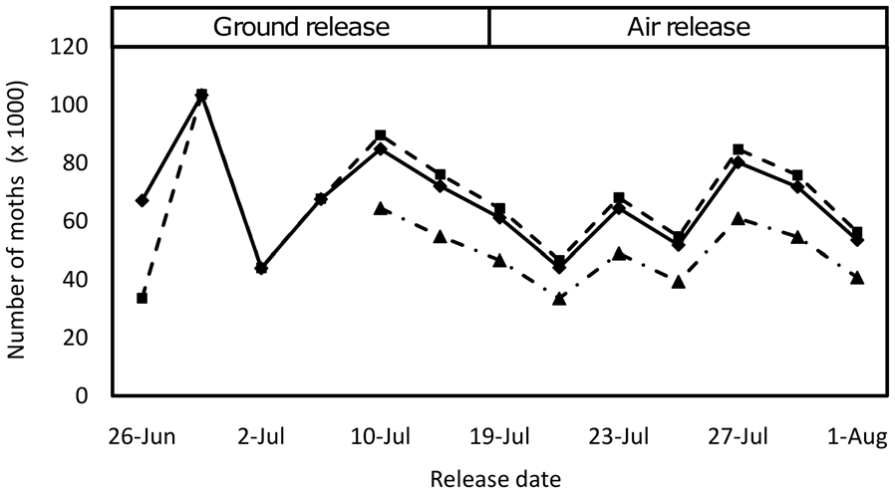
Number of moths of both strains released in Fields 1, 2 and 3 during the trial period. Field 1 = diamond data points with solid line; Field 2 = square data points with dotted line; and Field 3 = triangular data points with dotted line.

Moths were released in 2007 in three cotton fields in Yuma County, Arizona: Field 1, a 34.6-acre field cultivated following standard growing practices, except that no insecticide applications for any pests were applied; Field 2, a 36.4-acre field; and Field 3, a 26.4-acre field. Fields 2 and 3 were both cultivated with insecticide applications throughout the season for pink bollworm, Lygus, and whitefly control. Approximately 1.1 million moths of each type were released in total (Figure 3). After corrections for weight and mortality, moths were released at an equal release rate (numbers/acre) in all fields except for the first release date on 26 June when Field 1 received approximately double the moths of Field 2 (Figure 4). There were a total of 13 releases by ground and air between 26 June and 1 August for Fields 1 and 2. Field 3, which was added to the experiment on 10 July, received nine releases. The combined mean release rate for both moth types was 565 moths/acre/day. To estimate field persistence for each release type, trap monitoring continued after the last release until no further moths were recaptured for 14 days. Male moths were recaptured using gossyplure-baited traps. Analysis of pre-release samples indicated that, over the course of the release experiment, an estimated 552,000 APHIS males and 553,000 OX1138B males were released, a ratio of 0.501 to 0.499 (s.d. = 0.05). Analysis by paired *t*-test shows that this ratio is not significantly different to 1∶1 (*t* = 0.02, df = 24, p = 0.98). In addition, the proportion of released moths that were male was close to 0.5 for each strain (OX1138B: 0.51; 95% CI: 0.50–0.52; APHIS: 0.51; 95% CI: 0.46–0.55) and sex ratio did not differ significantly between the two strains (χ^2^ = 0.0114, df = 1, p = 0.91). The relative numbers of APHIS and OX1138B moths released was therefore considered to have been 1∶1 in all subsequent analyses.

**Figure 5:**
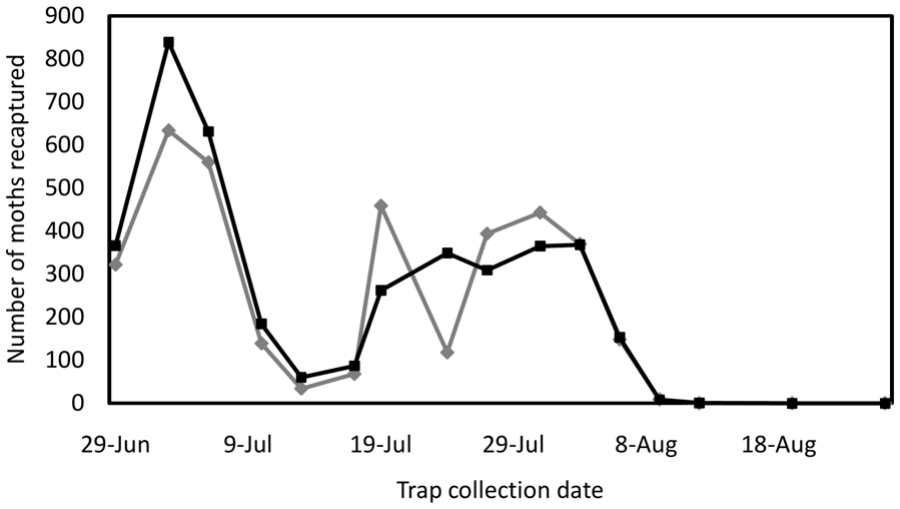
Number of OX1138B and APHIS male moths recaptured in all fields during the trial period. Black line = OX1138B; grey line = APHIS.

**Figure 6:**
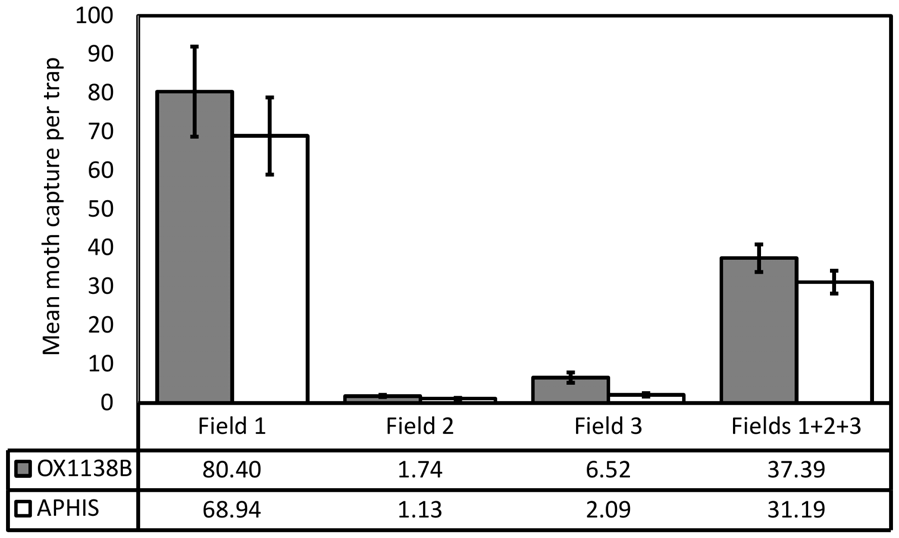
In-field recapture data during release period. Mean number of OX1138B and APHIS moths caught per trap is shown for each field separately, and for all three fields combined. Lower recapture rates in Fields 2 and 3 than in Field 1 were likely a result of pesticide treatment in the former. Filled bars = OX1138B, white bars = APHIS moths; error bars indicate Standard Error of Mean. The recapture rates of OX1138B and APHIS moths were significantly different (Poisson regression model, 95% CI: 7.8–33.3%; p<0.01).

To measure the accumulation of sexually active sterile males in the release area, traps were placed within the fields (Figure 5). These captured a mean of 37.4 OX1138B moths per trap (95% CI: 26.5–48.2) compared with 31.2 for APHIS moths (95% CI: 21.8–40.6). In other words, overall 20% more OX1138B moths were recaptured than APHIS moths (Figure 6), a significant difference (Poisson regression model, 95% CI: 7.8–33.3% more; p<0.01). Comparison of the recapture ratio of the two strains also showed significant variation between fields (i.e. the type by field interaction was also significant; χ^2^ = 7.81, df = 2; p = 0.02). In Field 1 overall 17% more OX1138B moths were recaptured than APHIS moths (Figure 6), a significant difference (Poisson regression model, 95% CI: 4.8–30% more; p<0.01). In Fields 2 and 3, where much fewer moths were recaptured, the figures were 53% more (95% CI: 37% less to 271% more) and 212% more (95% CI: 48–559% more), respectively.

**Figure 7:**
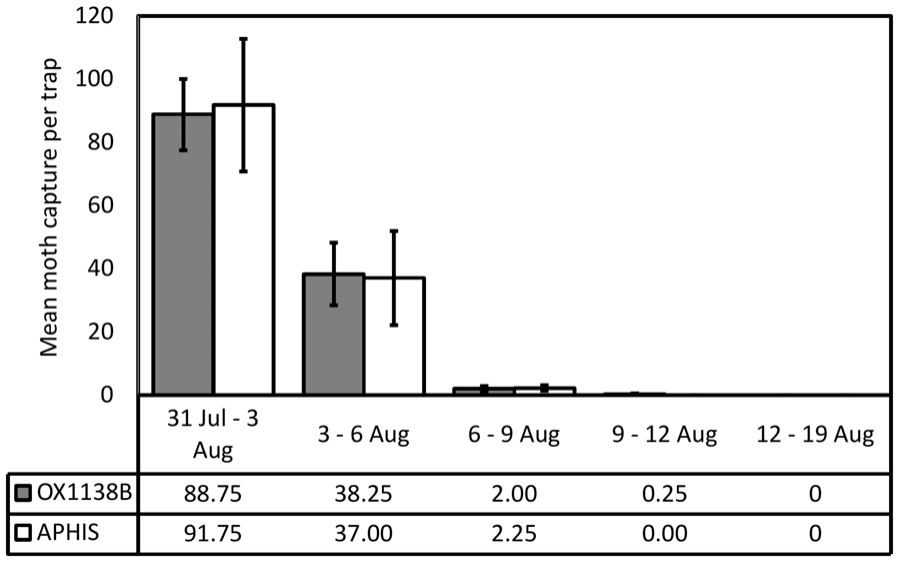
Moth persistence after end of release period. Trapping continued beyond the last release date of 1 August 2007, to assess the rate of decline of OX1138B and APHIS populations when no longer supplemented by additional releases. Mean number of moths caught within field 1 per trap are shown by date. Filled bars = OX1138B, white bars = APHIS moths; error bars indicate Standard Error of Mean. No significant difference in persistence between the strains was found (χ^2^ = 1.46, df = 1; p = 0.23).

After the last release of OX1138B and APHIS moths, five consecutive 3-day trapping periods were analyzed for the recapture of these moth types to assess the levels of persistence or residence time of released moths in the field (Figure 7). Reduced recapture over time likely relates to moth mortality, but could alternatively or additionally represent a reduction in female-seeking ability with time or dispersal beyond the trapping zone, and will therefore be referred to as residence instead of survival [17]. Residence was quantified by the estimated probability of daily persistence [18]–OX1138B, 54.2% (95% CI: 49.5–58.8 %); APHIS, 50.6% (95% CI: 49.2–51.7 %)–and average residence time–OX1138B, 1.63 days (95% CI: 1.42–1.88); APHIS, 1.46 days (95% CI: 1.41–1.51). There was no evidence for a significant difference in residence time in the field between the strains (χ^2^ = 1.46, df = 1; p = 0.23).

**Figure 8:**
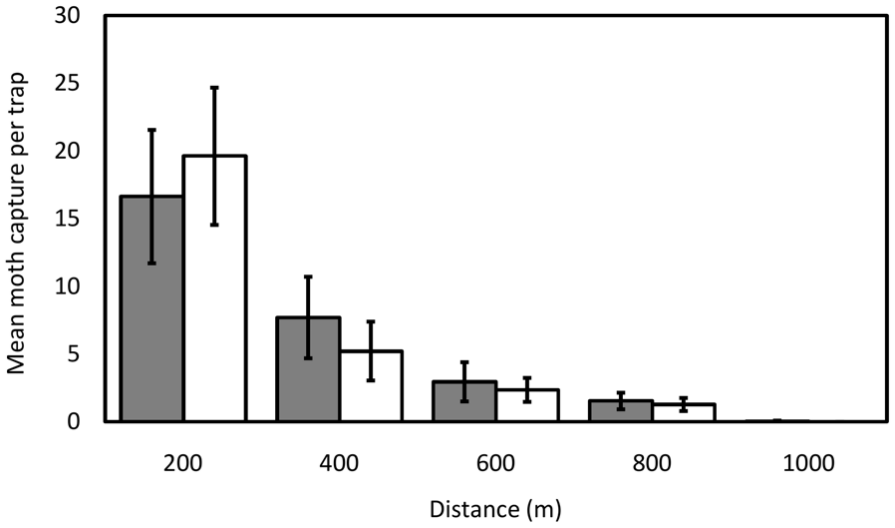
Dispersal of moths beyond release areas. Traps were set at 200 m intervals on cardinal axes outside Field 1. Mean numbers of OX1138B and APHIS moths per trap at each distance from field edge are shown. Filled bars = OX1138B, white bars = APHIS moths; error bars indicate Standard Error of Mean. There was a significant difference in the number of moths caught at different distances between the two strains (χ^2^ = 29.15, df = 4, p<0.01).

In order to observe and compare the dispersal of released moths, traps were also set at 200 m intervals on cardinal axes outside Field 1 up to a distance of 1000 m (Figure 8). There was a significant difference in the number of moths caught at different distances between the two strains (χ^2^ = 29.15, df = 4, p<0.01). Dispersal data were summarized in the form of mean distance traveled (MDT) and flight range within which 90% (FR_90_) of population dispersed [19], [20]. The MDT was significantly further for OX1138B (423 m) than APHIS (390 m) (χ^2^ = 5.68, df = 1, p = 0.02). The FR_90_ (OX1138B, 688 m; APHIS, 693 m) values were very similar.

Pheromone traps attract males that are searching for females, and are considered a good proxy for male sexual activity. We additionally used immobilized female moths to attract free-flying males. Wild type females (APHIS or University of Arizona (UoA), an independently derived strain) were placed in mating stations overnight with mating pairs observed and the males captured and analyzed for genotype (Table 1a). No significant difference was observed in the number of OX1138B and APHIS males attracted (χ^2^ = 0.00, df = 1; p = 1.00): 4.6% of females initiated mating with OX1138B males and 4.6% initiated mating with APHIS males.

**Table 1:** Moth mating performance.

(a) Wild type females with OX1138B or APHIS males			
Date	Female type and number	Mated with males	
		OX1138B	APHIS
7/26/07	APHIS (60)	3 (5.0%)	4 (6.7%)
8/3/07	UoA (60)	3 (5.0%)	1 (1.7%)
9/15/07	UoA (160)	7 (4.4%)	8 (5.0%)
Total	280	13 (4.6%)	13 (4.6%)

(b) OX1138B and APHIS females with wild males				
	OX1138B females		APHIS females	
Date	Number of females	Mated with wild males	Number of females	Mated with wild males
8/3/07	50	11 (22.0%)	50	13 (26.0%)
8/22/07	28	5 (17.8%)	34	9 (26.5%)
9/17/07	35	19 (54.3%)	36	21 (58.3%)
10/1/07	34	12 (35.3%)	34	11 (32.4%)
Total	147	47 (32.4%)	154	54 (35.8%)

Initiation of mating between (a) sentinel wild type [APHIS or University of Arizona (UoA)] female moths and released OX1138B, APHIS males or wild males present in the field; and (b) sentinel OX1138B or APHIS female moths and wild males. Initiation of mating was defined as a male and female joined together in the tail-to-tail position typical of mating in Lepidoptera. Wild males also initiated mating with the females in this experiment: three on 26 July, 2007, four on 3 August, 2007 and 59 males on 15 September, 2007 (the high number on this last date reflects the typical wild pink bollworm recapture in traps late in the growing season).

Sterile female moths may contribute to control by attracting wild male moths and ‘distracting’ them from mating with wild females, though the relevance of this is unclear as they presumably also attract sterile males [21], [22]. We tested the ability of OX1138B and APHIS females to attract males. Mating performance tests, in which immobilized female moths were used to attract free-flying males, did not demonstrate a significant difference in initiation of mating between OX1138B and APHIS females (χ^2^ = 0.09, df = 1, p = 0.76) (Table 1b). The mean percentage of mating initiation was 32.4% (95% CI: 16.3–48.4) for OX1138B females and 35.8% (95% CI: 20.8–50.8) for APHIS females.

In 2008, over 15 million OX1138B moths, also marked with Calco Red, were released during a SIT operational demonstration trial, in which the pink bollworm control program gained experience with the new moth strain by making releases and evaluating recaptures in a program control area. We took the opportunity to screen a sample of 92 trapped moths visually for the presence of Calco Red and DsRed2. Secondary screening was carried out by chromatography for Calco Red and by PCR for the presence of the OX1138B transgene. All 75 moths that scored positive for Calco Red were also positive for DsRed2 screening. This indicates that screening for DsRed2 is highly reliable. Furthermore, one OX1138B insect was screened as negative for the Calco Red marker, both visually and by chromatography, yet positive for DsRed2. PCR analysis confirmed the visual screening indicating that the moth was homozygous for DsRed2, as expected for a released sterile moth. Such a case, albeit from a limited sample size, highlights the potential for occasional incorrect classification of sterile moths as wild moths in the pink bollworm control program, and consequent unnecessary and costly response measures.

## Discussion

In the field, OX1138B performance was similar to or better than APHIS moths. Some measures of performance, such as recapture rate and dispersal, showed that OX1138B moth performance slightly exceeded that of APHIS moths. This is possibly a result of subtle differences in rearing conditions, although no significant differences in pupal weight or post-eclosion mortality were found between the two strains. It may alternatively reflect the different recent rearing histories of the two strains. The APHIS strain has been mass-reared continuously for more than 10 years. Continuous selection for traits such as short generation time may have inadvertently led to a slight loss of field performance. While OX1138B is derived from the APHIS strain, it was reared under more relaxed conditions for many generations before being mass-reared for the experiments described here. If differential rearing history is indeed the explanation for the better performance of OX1138B, this might argue for the use of a ‘filter rearing system’ [23], in which all mass-reared insects are recently derived from a relatively small mother colony. Such systems were designed to allow rearing of strains with unstable, non-wild type genetics and could therefore be useful were the OX1138B strain to be found to break down at a significant rate, a hypothetical event that has not been detected to date. A filter rearing system would also allow moths to be reared under relaxed conditions and only reared at high density for a few generations before release. However, any potential benefit in terms of field performance would have to be weighed against the additional rearing costs involved.

The ability of a moth strain to compete with wild moths and its relation to SIT efficacy is difficult to determine. The United States Department of Agriculture, Animal Plant Health and Inspection Service (APHIS) along with cotton industry cooperators in several states and the government of Mexico , however, have an effective ongoing SIT program against pink bollworm [3], [24]. We therefore assumed that the wild type ‘APHIS’ strain used in this program is of adequate performance, albeit not necessarily optimal, in respect of key parameters such as survival, dispersal and mating competitiveness. We used this strain background for transformation with OX1138, and compared the performance of the resulting OX1138B insertion strain with that of the wild-type APHIS strain. Our data show that attributes critical to SIT in the field–ability to find a mate and to initiate copulation, as well as dispersal and persistence in the release area–are comparable between OX1138B and APHIS. These results imply that OX1138B would provide similar effectiveness to APHIS if it were substituted in the SIT program. In addition, more consistent and reliable identification of released moths with a marker that provides an extra degree of certainty over current methods would provide economic savings and greater confidence in the decision-making process to initiate quarantine or enhance control measures. This will become more valuable as the control program progresses with pink bollworm eradication in the southwestern USA. As wild population levels decrease to near zero, the area around a wild moth capture will be heavily treated with sterile moths and other interventions. At this stage, mistaken identification of a released sterile moth as a wild moth would be particularly costly.

A further benefit of the DsRed2 marker is that it is heritable, unlike Calco Red. This would facilitate the use of F_1_ sterility for the SIT, with the advantage of lower radiation doses, and therefore better performing insects [25]. A heritable marker is just one of several potential improvements that transgenic technology can offer insect control programs [26], [27]. For example, genetic technology exists to allow male-only release, or to obviate entirely the need for irradiation in SIT using the concept of autocidal biological control or genetic sterilization [21], [26], [28]–[30]. Several groups are trying to develop strains and strategies to convert wild populations of mosquitoes to a less harmful form, for example with reduced ability to transmit a given pathogen (‘refractory insect strategy’) [27], [31]–[33]. These strategies all depend on engineered insects competing for mates with wild insects in the field. The first genetically engineered strain to be tested in this way performed well relative to a standard strain, which is encouraging for the whole field of genetic control.

## Materials and Methods

### Pink bollworm strains

The APHIS wild type strain, derived from wild insects caught in Arizona, USA, has been mass-reared, sterilized and released for the SIT in the USA and Mexico since 1996. The OX1138 construct comprises a fluorescent protein marker cassette, flanked by the transposable sequences from the *piggyBac* transposon. Expression of the DsRed2 fluorescent protein is regulated by *Opie2*, a promoter fragment from the *ie2* gene of baculovirus *Orgyia pseudotsugata* nuclear polyhedrosis virus, a pathogen of the Douglas-fir tussock moth (*O. pseudotsugata*). To build this construct, a previous plasmid from our laboratory-OX513 [34]-was cut with restriction enzymes *Not I* and *Pac I* to remove the cassette containing the Act5C promoter and DsRed2, which was replaced by the *Opie2* promoter fragment with DsRed2. This intermediate plasmid was cut by *Asc I* and *Xba I* to remove the teto-tTAV sequences; the remaining fragment was blunt-ended, self-ligated and transformed to *Escherichia coli*. The final construct was verified by restriction digestion. Transgenic strains of DsRed2 pink bollworm were generated by *piggyBac*-mediated germline transformation of the APHIS strain with OX1138, using a standard micro-injection procedure [35]. Four independent insertions of OX1138 resulted from these injections: OX1138A-D. The C and D insertions appeared to be associated with recessive lethal or semi-lethal insertions. OX1138A homozygotes seemed to have low fecundity. A homozygous line of OX1138B was generated, confirmed over several generations by genetics and PCR. This strain was transferred to the APHIS-PPQ-CPHST quarantine rearing facility in Phoenix, AZ. OX1138B had been reared under these simulated mass-rearing conditions for approximately 12 generations prior to the commencement of the 2007 experiment.

### Cage trials

The field cage tests (USDA permit number 06-150-01r) compared OX1138B (100 Gy gamma radiation dose) and APHIS (100 Gy or 200 Gy). Before eclosion, pupae were marked by applying fluorescent powders (‘Light Green’, ‘Rocket Red’, ‘Light Orange’ and ‘Blue’), which adhered to the moths when they eclosed. A different powder color was used for each moth type. Moths were irradiated at the appropriate dose and co-released in screened quarantine cages (3 m×3 m×2.5 m), outdoors, which contained mature cotton plants. One gossyplure-baited delta trap was first set at canopy height in each cage 3, 6 or 9 days after moths were released. Recaptured moths were screened for the fluorescent powder and DsRed2 marker every day thereafter until moth recaptures stopped.

### Insect rearing

For field experiments, APHIS moths were taken from the pink bollworm mass-rearing facility (MRF) at the pupal stage and thereafter maintained and processed in parallel with OX1138B in a dedicated quarantine module. All rearing procedures followed standard protocols for rearing pink bollworm [36]: egg collection cages with 35 g pupae and automated scale collection; egg pads from each cage are divided into eight equal pieces (each holding approximately 4000 eggs) and used to ‘infest’ 250 g of artificial diet. OX1138B was reared in a dedicated quarantine module. Mass-rearing was identical except that OX1138B rearing containers were put into 2.8-litre tubs during the ‘cut-out’ stage (when final-instar larvae exit diet) as an additional measure of quarantine security, thereby preventing larvae from wandering and pupating away from the Hexcel pupation substrate. For rearing purposes, mature pupae were loaded into the eclosion system, which consisted of the same equipment used by the MRF: eclosion boxes, collection lines with ultra-violet fiber-optic light source, cyclone knockdown traps and a 3°C collection chamber. Batches with 4000 g of pupae destined for sterilization and release as adults were loaded into the adult collection system. Pupae of each type placed into the collection system were selected to closely match in age and maturity. Moth collections were made twice daily and stored in trays at 3°C until completion of quality control sampling and irradiation was conducted.

### Moth sterilization

Moths aged up to 36 h post-eclosion were used for the release experiment. A sample of 100–200 moths was collected from each collection period. The mean weight, mortality rate and sex ratio were measured to estimate the total number of male and female moths in each collection. The total number of moths for release was adjusted based on moth weight and mortality for each collection period with the aim of releasing the same number of APHIS and OX1138B moths of the same post-eclosion age. Moths were packaged for release in irradiation canisters, transported to the irradiator in an ice chest maintained at 4°C, gamma-irradiated at 200 Gy, and held at 4°C until release.

### Moth release

Releases of APHIS and OX1138B moths were carried out in Yuma county, Arizona, in 2006 (USDA permit number 06-163-01 r) as a preliminary experiment, prior to larger-scale releases in the same region in 2007 (USDA permit number 07-015-102 r) and 2008 (USDA permit number 08-105-102 rm). In 2007, releases of sterile moths took place in three fields of conventional, non-*Bt* cotton, in Yuma County, Arizona. Delta Traps (Scentry Biologicals Inc.), baited with 2 mg of the synthetic female sex pheromone, gossyplure, were placed every 3–7 days, depending on recapture rates. Trap height was set at canopy level, and traps were distributed at approximately one per 9 acres (four traps in fields 1 and 2; three traps in field 3). Two extra traps were set on the north and south field edges of fields 2 & 3 in case insecticide application prevented trap distributors and collectors from entering the field. To estimate if there were differences in dispersal for field 1, additional traps were set up outside of Field 1 at 200 m intervals in each cardinal direction up to 1000 m from the field edge. Releases were made at regular intervals between 26^th^ June and 1^st^ August 2007. They were carried out 3–4 times per week by two methods: (a) hand release, spreading pre-mixed moths of both types along entire cotton rows separated by 50 m distances (26^th^ July until 10^th^ July); and (b) air release, following standard program equipment and release protocols (17^th^ July until 1^st^ August). In 2008, sterile OX1138B moths were released by air over ∼2500 acres of cotton (2382 acres of *Bt* cotton and 174 acres of non-*Bt* cotton) in Yuma County, at a rate of 1–2 million moths per week (from 11^th^ June until 5^th^ September). Unlike the 2007 trial, which was designed to provide a direct, side-by-side comparison of OX1138B and APHIS, only OX1138B moths were released over this area. Releases were carried out three times weekly, using moths up to 48 h old. Delta traps were deployed in release areas following the standard program rate for Yuma of one trap per 60 acres. The intention was to follow normal program operations, with the addition of DsRed2 screening and molecular analysis. Prior to the commencement of OX1138B releases, APHIS strain moths were released in the area in accordance with eradication program protocols.

### Trap collection and analysis

Traps were evaluated within 2 weeks after collection. Trapped moths were screened using bright field and fluorescence microscopy and scored according to the following criteria: moths with Calco Red dye only were scored as APHIS moths; moths with Calco Red plus DsRed2 fluorescence were scored as OX1138B moths, and moths showing neither Calco Red nor DsRed2 fluorescence were scored as wild moths.

### Validation of trap reading

To provide validation of visual screening for DsRed2 fluorescence in recaptured OX1138B moths, 32 traps were screened by microscopy (as above) and the number of Calco Red-positive and -negative moths recorded. DNA was then isolated from the moths (Nucleospin® Tissue, Macherey Nagel) and genotyped within 14–66 days of trap collection by PCR to amplify two genomic sequences: that spanning the junction of the transgene and the adjacent genomic sequence, to detect the presence of the transgene insertion; and the sequence at another genomic location (no transgene), to act as a genomic DNA control. For the former reaction, DNA extracted from an OX1138B moth would result in an amplified fragment of 580 bp. The absence of this fragment, together with a positive DNA control PCR (336 bp) indicated an APHIS or wild moth.

### Mating performance

In 2007, the mating performance of OX1138B and APHIS moths was tested by observing the matings between sentinel female moths at mating stations and male moths present in the field. Two tests were carried out: (i) assessment of the mating performance of female OX1138B and APHIS moths, irradiated at 200 Gy, with wild male moths present in the field; and (ii) assessing the performance of released male OX1138B and APHIS moths, irradiated at 200 Gy, with sentinel un-irradiated wild females at mating stations. OX1138B and APHIS female moths were collected from the lab reared colony as pupae and placed individually in vials for eclosion. Upon eclosion, moths were fed sugar water (7.5% sucrose, 0.067% methyl p-hydroxybenzoate) and maintained in a 12∶12 L:D light cycle until field testing began. Prior to placement in the field, one wing of each female was clipped (to prevent escape) and the moth was irradiated at 200 Gy. They were maintained at 12–18°C during transport to the field. Mating stations were set up using a 2.8-litre paper carton bucket set on a 1 m stake at 1 m height, which was the average canopy height of the cotton field. To prevent the sentinel moths from crawling out, the buckets were painted with a 1 cm band of Fluon® paint near the top of the inside rim. Mating stations were set at canopy height within cotton rows at 10 m intervals. At dusk, a single moth was placed in each bucket along with a cotton leaf to provide shelter. Once females began calling and males started responding to the traps, mating stations were visited every 30 min throughout the night until mating ceased. A female observed in copula with a male moth was counted as a successful mating. All mating pairs were collected with an aspirator and placed on ice for transport to the laboratory where the male from each mating pair was inspected by bright field and fluorescence microscopy to determine its identity: OX1138B, APHIS or wild. The percentage of mating success for each female type was calculated as the proportion of total number of sentinel moths at mating stations that mated. This experiment was replicated on four nights from August to early October 2007. Testing the mating success of OX1138B and APHIS males were conducted using similar procedures as testing females. For this experiment, the sentinel females were derived from recently colonized wild populations maintained at the University of Arizona, except for the first replicate where lab-reared APHIS females were used. Females were handled as described above, except they were not irradiated. This experiment was replicated three times from late July to mid-September 2007.

### Statistical analysis

#### Cage trials, 2006

For comparing pupal weights, we tested the three groups (OX1138B 100 Gy, APHIS 100 Gy and APHIS 200 Gy) using a three-way Kruskal-Wallis test. If this was significant, two-way Kruskal-Wallis tests were conducted to identify where differences lay between the moth types. For recapture rates, logistic regression models were conducted for total recaptures (with effects for group, day of first trapping and cage) and for captures on the first day of trapping only.

#### Quality of mass-reared pink bollworm

The data on mean adult weight and mortality of APHIS and OX1138B were analyzed using the sign test (http://www.graphpad.com/quickcalcs/binomial1.cfm).

#### Sex ratio of released moths

The sex ratio of subsamples from all batches of moths collected (n = 28) were recorded from which the means, variance and 95% confidence intervals were calculated. Chi-squared tests were conducted to test the null hypothesis that sex ratio was the same for APHIS and OX1138B moths.

#### Recapture

Transect traps were omitted from this 2007 analysis, as they were only available for Field 1. The remaining data from each field were compared to estimate relative numbers recaptured within the three fields (1, 2 and 3). Poisson regression models were constructed with effects of: time period, trap number, a field by time period interaction, and type (OX1138B or APHIS). To allow for any overdispersion (or extraPoisson variation, that is unmodeled variation beyond that expected from a Poisson distribution), we used an overdispersion factor (the square root of the model deviance divided by its residual degrees of freedom) to expand standard errors of parameter estimates appropriately. The effects other than “type” were included to adjust for nuisance sources of variation that might obscure the comparison of interest. If the “type” effect was significant then a test was carried out to determine if there was a “type” by field interaction. Sixteen time-periods for this analysis were identified: traps collected on 29 June, 3, 6, 10, 13, 17, 19, 24, 27, 31 July, 3, 6, 9, 12, 19, 26 August. There were insufficient data from traps collected after 6^th^ August so data from these collections were omitted.

#### Persistence

The probability of daily persistence (PDP) was estimated from the regression of log_10_ (recaptures +1) against recapture time where the antilog of the slope of the regression line is the PDP [18]. Average residence time (ART) was derived from the PDP using the equation: ART = 1/-Log_e_PDP [17]. Confidence intervals for PDP and ART were derived using bootstrapping (based on Poisson distributions). For analysis for a difference in residence time between the strains, only traps collected before 9^th^ August 2007 were included, as traps collected on or after this date contained relatively few moths.

#### Dispersal

These analyses were based only on data from the transect traps. A standard contingency-table chi-squared test was used to test for an association between the type (APHIS or OX1138B) and the distance trapped (200, 400, 600, 800 or 1000 m). The null hypothesis of no association is equivalent to the hypothesis that the two types dispersed equally. The mean distance traveled (MDT) was calculated for each type using the method of Lillie [19] and Morris [20], with the delta method used to derive the variance for this statistic. Based on the estimates’ means and variances, a chi-squared statistic was used to test the null hypothesis of the mean distance traveled being equal for the two types. The flight range statistics (within which 90%-FR_90_-of population dispersed) were calculated using the regression method of Lillie [19] and Morris [20] in which the log-transformed trap distance was regressed against the cumulative number of expected recaptures.

#### Mating performance

The data were analyzed by a chi-squared test comparing the total mating success of APHIS and OX1138B moths over all of the nights, to determine whether there was a significant difference in mating success between the two moth types that mated.
